# Supplementary figures and images for: Escherichia coli ZipA Organizes FtsZ Polymers into Dynamic Ring-Like Protofilament Structures
Source: mBio. 2018 Jun 19;9(3):e01008-18. doi: 10.1128/mBio.01008-18 (PMC6016244; doi:10.1128/mBio.01008-18)

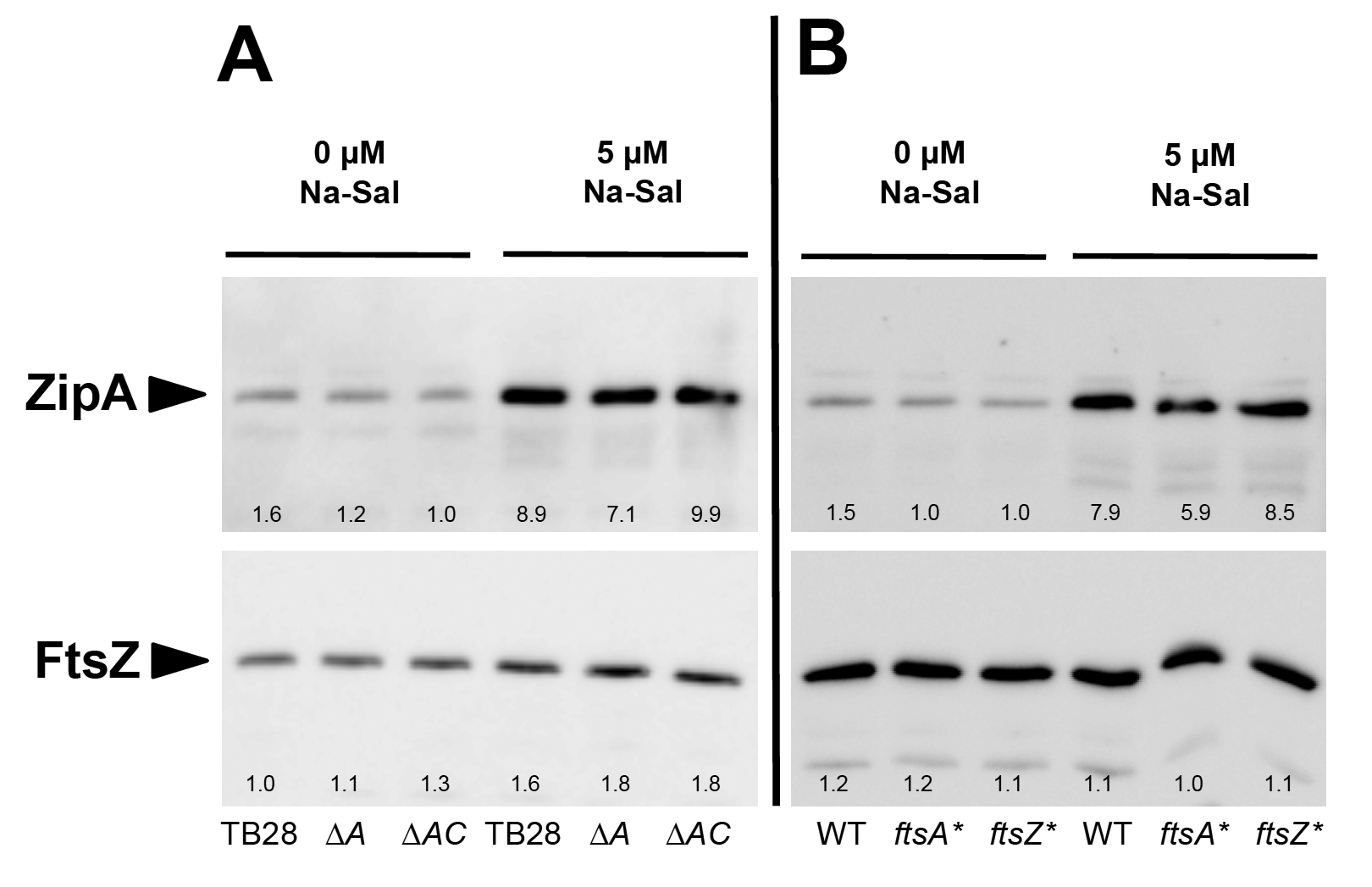

Supplement: FIG S1 [file mbo003183921sf1.tif]

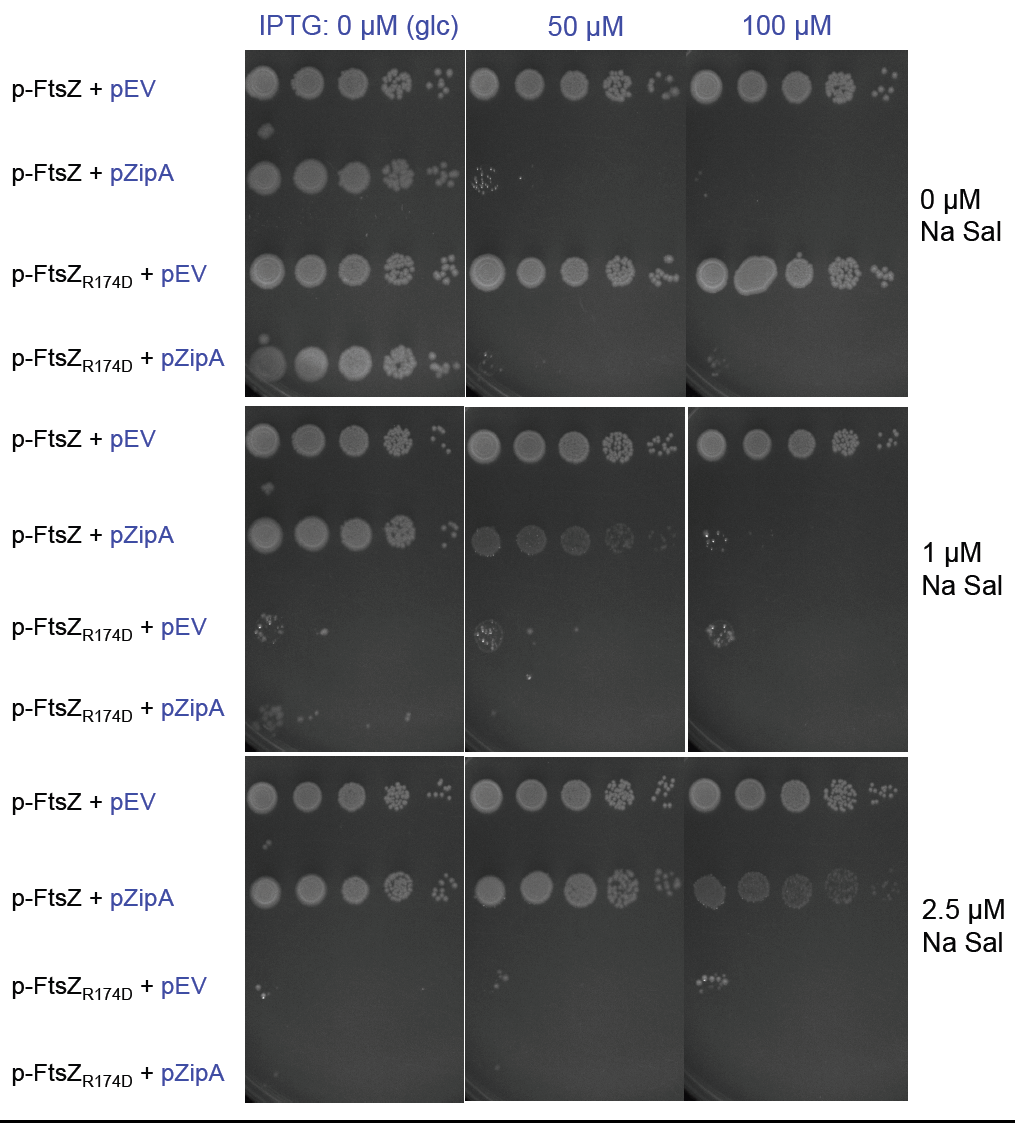

Supplement: FIG S2 [file mbo003183921sf2.tif]

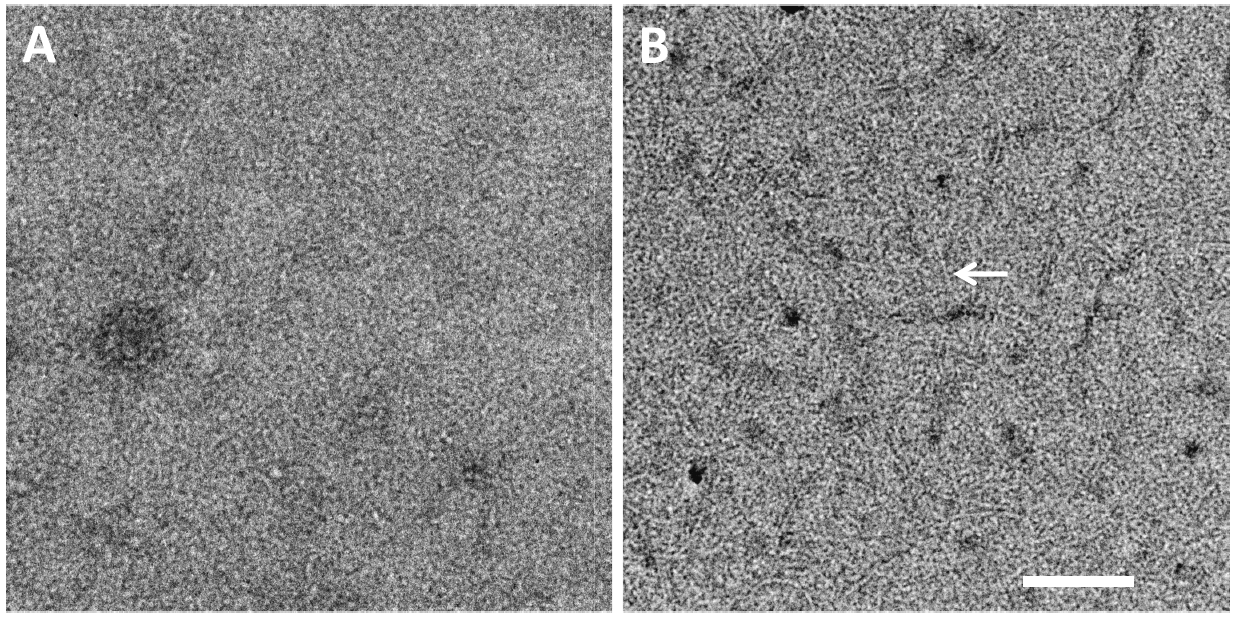

Supplement: FIG S3 [file mbo003183921sf3.tif]

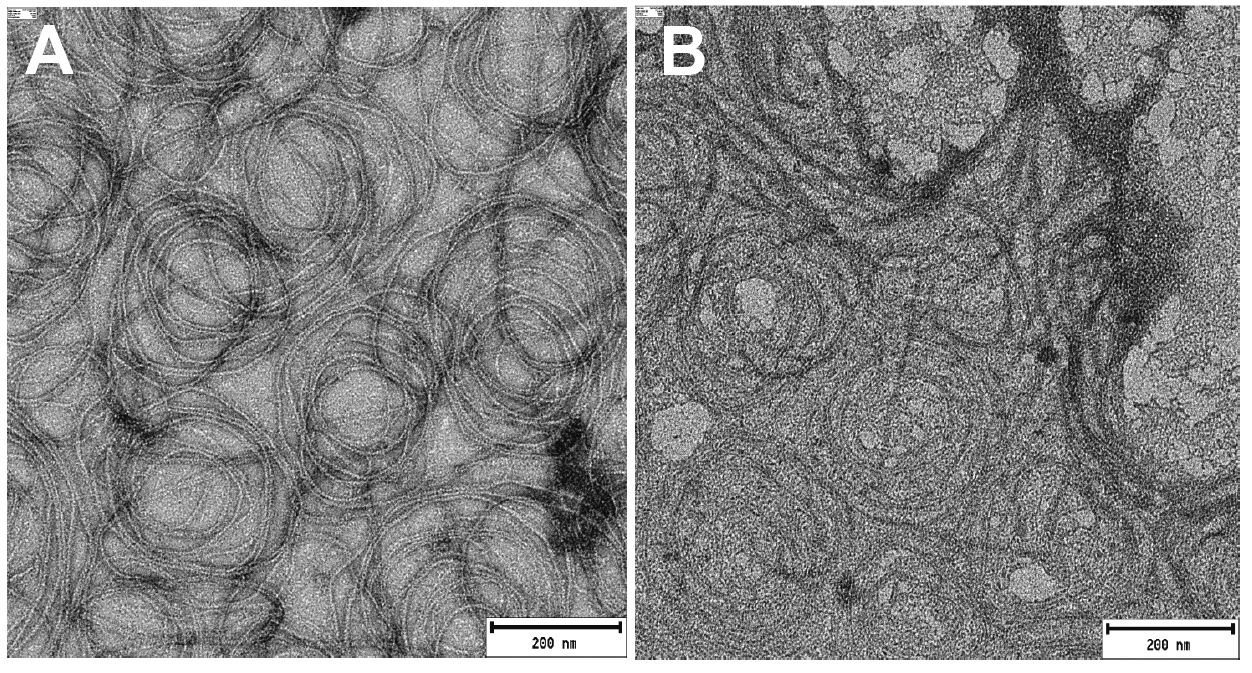

Supplement: FIG S4 [file mbo003183921sf4.tif]

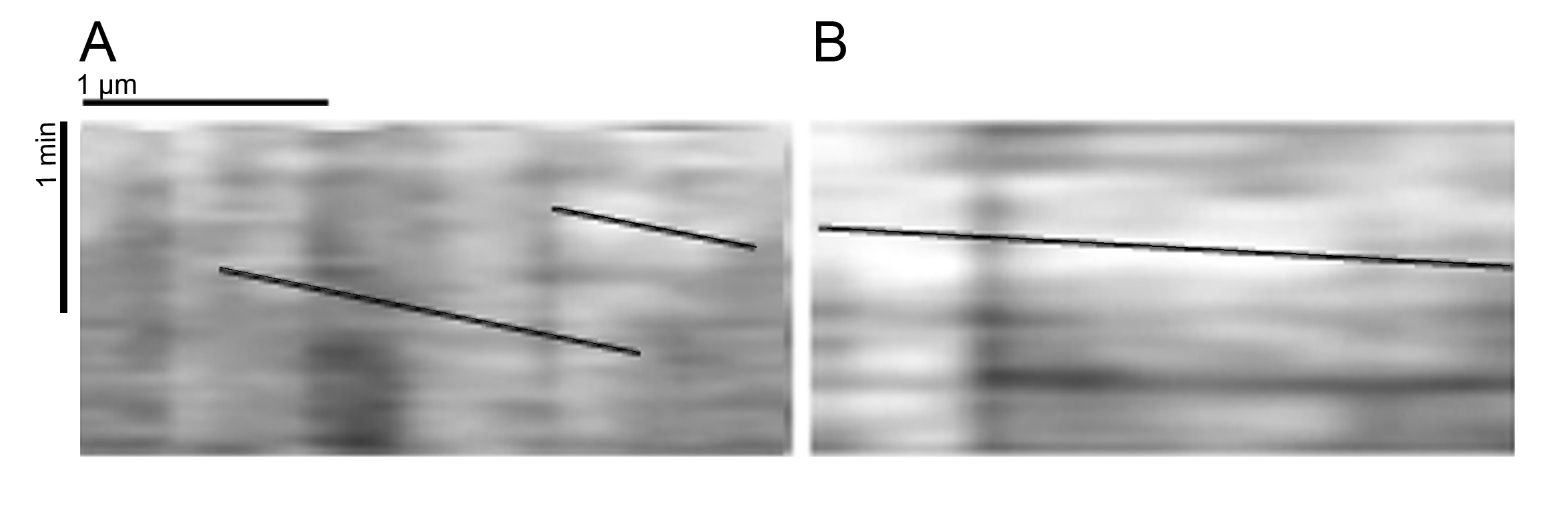

Supplement: FIG S5 [file mbo003183921sf5.tif]
